# Is the Goat a New Host for the G3 Indian Buffalo Strain of *Echinococcus granulosus*?

**DOI:** 10.1100/2012/286357

**Published:** 2012-05-15

**Authors:** Pietro Calderini, Simona Gabrielli, Gabriella Cancrini

**Affiliations:** ^1^Sezione di Rieti, Istituto Zooprofilattico Sperimentale Delle Regioni Lazio e Toscana, V. Tancia 21, 02100 Rieti, Italy; ^2^Dipartimento di Sanità Pubblica e Malattie Infettive, Sapienza Università di Roma, P. le Aldo Moro 2, 00185 Roma, Italy

## Abstract

Four goats bred in Central Italy (province of Rieti) revealed, in the liver, metacestodes of *Echinococcus granulosus*. The cysts, unilocular and fertile, were examined by microscopy and molecular diagnostics. Morphological data on the rostellar hooks are in agreement with the original description of the strain found in buffaloes and are largely compatible with those reported in Europe for cattle and humans. Specific PCR followed by DNA sequencing of the mitochondrial cox1 gene revealed for all the isolates 99.5% identity to the reference strain G3 genotype and 99.3% and 99.1% to G2 and G1, respectively. Further genetic markers (nad1 and 12S rRNA) confirmed the identity of the goat isolates to the G3 strain. This genotype, here reported for the first time in goats, proved to have a wider than previously supposed host range, therefore its relevance in human hydatidosis is expected to be more often evidenced.

## 1. Introduction

Cystic echinococcosis due to *Echinococcus granulosus* is regarded as emerging or reemerging zoonosis also in countries of the Mediterranean basin. High endemicity of this parasite depends on extensive sheep farming; in fact, it is mainly transmitted in a cycle between dog definitive host that harbours the small intestinal tapeworm and livestock (especially sheep) after the latter ingests the microscopic eggs while grazing pastures that are contaminated with dog faeces. However, several other domestic and wild herbivores/omnivores and carnivores (like wolf and fox) can be involved in the parasite transmission [1]. Humans became exposed to the eggs of the tapeworm after close contact with an infected dog or, more often, after direct/indirect contact with its contaminated environment.

The wide host range and further differences in biology and geographical distribution may be related to the existence of an *E. granulosus* complex of species, as suggested by genetic studies that have evidenced a number of genetic variants [2]. Currently, *E. granulosus sensu lato* has been split in *E. granulosus sensu stricto* (genotypes G1–G3, which also includes the lion strain *E. felidis*), *E. equinus* (genotype G4), *E. ortleppi* (genotype G5), and *E. canadensis* (genotypes G6–G10) [3].

This categorization follows the pattern of biological characteristics (life-cycle pattern, host specificity, development rate, pathogenicity, antigenicity, sensitivity to drugs, transmission dynamics, epidemiology, and control of echinococcosis/cystic hydatidosis also in humans). Therefore, detailed analysis on the strains found is crucial to define biological and pathological characters of each of them and to recognize genotypes of zoonotic interest.

As for Italy, during a survey on bovine hydatidosis in Central Italy (Lazio region, province of Rieti) we recovered the common G1 sheep and the G3 Indian buffalo strains of *E. granulosus* in cattle [4] so suggesting a wider than previously supposed host range and geographical distribution of the G3 genotype. This paper aims to also report, for the first time, the occurrence of the G3 genotype in four goats (two females —4 and 5 years old—and two males—3 and 4 years old—all bred in the wild) living in the same area.

## 2. Materials and Methods

### 2.1. Macro- and Microscopical Examination

Four hydatid-like cysts were removed from the liver of four goats, measured, and examined by microscopy to check for the presence of protoscolices in the fluid filling and on the cystic membrane and then to analyse the morphometric characteristics of the rostellar hooks. Protoscolices were mounted in polyvinyl lactophenol, and sufficient pressure was applied to the cover slip to cause the hooks to lie flat. The number, shape, and arrangement of rostellar hooks were assessed, and several components of both large and small hooks were measured (number of hooks, total length, and blade length) on the basis of studies that indicated these parameters as valid for identifying *E. granulosus* strains [5]. Measurements were made using a calibrated eyepiece micrometer under oil immersion. Part of the sample was used for biomolecular analyses aimed to assess the genotype of the isolate.

### 2.2. Molecular Analyses

Genomic DNA was extracted (Wizard SV Genomic DNA Purification Kit, Promega, USA) from each hydatid cyst material (protoscolices and germinal layer), and the cytochrome c oxidase subunits 1 (cox1) gene was PCR-amplified. In addition, target sequences of the mitochondrial DNA coding for nad1 (NADH dehydrogenase subunit I) and 12S rRNA were also amplified, according to protocols previously described [6, 7]. Positive amplicons were gel-purified (NucleoSpin Extract, Macherey-Nagel Inc., Bethlehem, PA, USA) and directly sequenced. The obtained sequences were aligned using ClustalW with available sequences for the *E. granulosus* genotypes.  Table 1 summarizes genes, primers, sequences used for comparisons and corresponding accession numbers in GenBank.

## 3. Results

### 3.1. Macro- and Microscopical Examination

**Figure 1:**
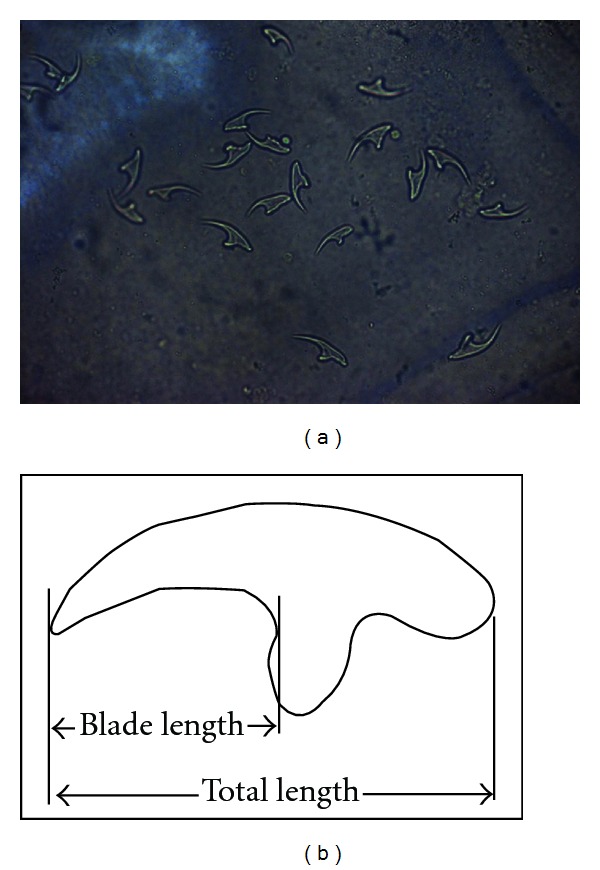
(a) Some large and small hooks isolated from the rostellum. (b) Diagram of measurements made on protoscolex rostrellar hooks (from [5]).

Morphological analysis of the cysts removed from the liver (one from each animal) revealed that they were subspherical in shape, 3–5 × 4–6 cm, unilocular, fluid-filled, and containing a different number of protoscolices (one, two, three, two) (Table 2). The rostella consisted of two rows of alternating large and small hooks (34–37 in number); large hooks were 25–27 *μ*m in total length and 12-13 *μ*m in blade length, whereas small ones were 19–22 *μ*m in total length and 9–12 *μ*m in blade length.  Figure 1 shows some large and small hooks (a) isolated from the rostellum and in preparation for counts and measurements of the parameters considered as valid for identifying *E. granulosus* strains (b).

### 3.2. Molecular Analyses

Molecular identification proved the strains involved in the infection to be highly identical to the G3 buffalo strain. In fact, the analysis of the variable sites of the cox1 sequences obtained for the four samples indicates 99.5% identity to G3 and 99.3% and 99.1% to G2 and G1, respectively (Table 3). The genetic identity to the other recognized species was lower: 93.4% to* E. equinus*, 92.9–92.7% to *E. canadensis*, and 92.7% to *E. ortleppi*. Sequencing of the nad1 and 12S rRNA supports these results. Blast identity search evidenced higher identity to the G3 (99.78% and 99.64%, resp.) than to G1 strain (99.30% and 98.93%, resp.).

## 4. Discussion

To the best of our knowledge, strains to date reported in goats are the sheep strain G1 (widely distributed), the cattle G5 and the pig G7 strains (present also in Europe), and the camel strain G6, probably absent from Europe [6, 8–10]. Thus far the G3 genotype, the “Indian buffalo strain,” has been further found by us in Italy in a few sheep and cattle in Lazio [11], in only one cow in Abruzzo [12], in cattle and water buffaloes in Campania [13–15] and in bovines of the same area where these positive goats were bred [4], but never in goats. Hence, the G3 genotype confirms an extra-India geographical distribution and in Italy demonstrates that other animals are suitable hosts and can act as intermediate hosts. Even people are included among the G3 strain hosts [16]; therefore this genotype could be more often evidenced in human hydatidosis, and the importance of genotyping the isolates of *E. granulosus* has to be stressed in order to assess the contribution of each strain to the epidemiology of human hydatidosis.

The amount of genetic variation among the three genotypes belonging to *E. granulosus s.s.* is very low, and it has been hypothesized that the G3 Indian buffalo strain may be a variant of the common sheep strain G1 or closely related groups. However, it has been demonstrated that, even if cox1, rrnS, nad1 genes [17] and heteroduplex comparison of a microsatellite from the U1 snRNA genes [18] fail to differentiate G1 and G2 strains, the first two aforementioned genes point out a significant genetic differentiation between G1 and G3 genotypes, with fixed nucleotide substitutions, and allow their discrimination [7]. To rapidly differentiate G1 from G2/G3, a real-time PCR protocol that uses as marker the 12S mtDNA gene has been recently designed [19], which, however, do not evidence mutation between G2 and G3 [16].

Therefore, it seems that G1 and G3 can be considered different strains, but available data are not conclusive and our findings cannot help. In fact, the very high genetic identity to the G3 strain of the goat isolates we found using the available more efficient molecular analyses and the morphology of the hooks, which are in general agreement with the original description [8] of the species found in buffaloes (24–34 *μ*m and 18–30 *μ*m), unfortunately are from only four cysts. If compared with morphometric data of protoscolices of *E. granulosus* from Europe [5] large hooks are more related (in total length) to that of cattle and humans, but they fit in partly (blade length) with those reported for sheep; small hooks are shorter than those of sheep and their blade (longer than in sheep) is more related to that of cattle and horse. Therefore, genetic and morphologic data on the goat isolates support the identification of the parasite found in this animal as G3 rather than as G1.

## 5. Conclusions

This is the first report of the G3 Indian buffalo strain of E. granulosus in the goat. Until G3 remains a distinct strain, as supported by recent observations [7, 19], goats have to be regarded as possible suitable hosts.

##  Authors' Contribution

G. Cancrini conceived the study, participated in its design, and helped to draft the paper. P. Calderini gave substantial contribution to conception, design and coordination of the study and carried out parasitological analyses. S. Gabrielli carried out the morphological analysis of the hooks collected and the molecular genetic studies and participated in the interpretation of the results.

## Figures and Tables

**Table 1:** Genes, primer sequences, and accession numbers used for genetic comparisons.

Genes	Primers	Sequence (5′–3′)	Accession numbers
cox1	JB3	TTTTTTGGGCATCCTGAGGTTTAT	G1: DQ062857
	JB4.5	TAAAGAAAGAACATAATGAAAATG	G2: M84662
			G3: M84663 G4: M84664 G5: M84665 G6: M84666 G7: M84667

12S	p60	TTAAGATATATGTGGTACAGGATTAGATACCC	G1: AY462129
	p373	AACCGAGGGTGACGGGCGGTGTGTACC	G3: DQ822451

nad1	JB11	AGATTCGTAAGGGGCCTAATA	G1: DQ856470
	JB12	ACCACTAACTAATTCACTTTC	G3: DQ856469

**Table 2:** Main features of the hydatid cysts found in goat liver.

Animal no.				
	1	2	3	4
Cyst size (cm)	3 × 4	4 × 6	3 × 5	5 × 6
Protoscolices present	1	2	2	3
No. of hooks	34	34–35	34–35	34–37
Arrangement of hooks	Large and small hooks alternating in 2 rows			
*Large hooks: *				
Total length (*μ*m)	25	25	26	27
Blade length (*μ*m)	12	12	13	12
*Small hooks:*				
Total Length (*μ*m)	20	19	22	21
Blade length (*μ*m)	10	9	12	11

**Table 3:** Alignment of the variable sites in the partial cox1 mitochondrial gene of the isolates of *E. granulosus sensu lato* evidenced in the goats (goat 1–4) examined in Central Italy with available sequences for other genotypes deposited in GenBank (E.g1–7).

Alignment positions					
		1111111	1111222222	2222222233	3333333333
	11333455	5670011244	5579123444	5556788900	0111233445
	3635369316	7685814347	3945687058	6787623703	6258169582
GOAT1	TAAAGTTGTC	GTGGTCGTGG	GGCGGAGGCG	GCTGATGGGT	GGATGTCGAT
GOAT2	……….	……….	……….	……….	……….
GOAT3	……….	……….	……….	……….	……….
GOAT4	……….	……….	……….	……….	……….
E.g1	……….	.C……..	……….	.T……..	……T..A
E.g2	………T	……….	……….	……….	……T..A
E.g3	……….	……….	……….	……….	……T..A
E.g4	.G..AG..GT	…AAT.G..	T.TATG.A.A	AAGT.G.T..	TA..T.TAGA
E.g5	.GGT..C.GT	T.AA.TT…	..TT.GA.TA	CG.TG.ATAC	.TT.TGT.GA
E.g6	..GT.G.TGT	T..A.TTAAA	.TTT.GA.TA	…TG.ATA.	..CCT.T.GA
E.g7	C.GT.G.TGT	T..A.TTAAA	.TTT.GA.TA	…TG.ATA.	..CCT.T.GA
